# Texas Sets the Pace in Railway Car Sanitation—Dr. Johnson’s Bill

**Published:** 1903-04

**Authors:** 


### TEXAS SETS THE PACE IN RAILWAY CAR SANITATION—DR. JOHNSON'S BILL.

We got knocked out in our efforts to secure a law giving some one authority to institute sanitary measures throughout Texas for the prevention of other diseases than those now guarded against by quarantine, but Texas is the first State in America to pass a law requiring railway companies to disinfect their coaches. The substance of this bill was embraced in the two health bills that met their death at the hands of our friends. Being defeated there, it was made a separate bill, drawn jointly by Drs. Johnson and Tabor. The committee as a committee had nothing to do with it. The honor of it belongs to the Honorable Jno. M. Johnson, Representative from Lee County. It is a plume in his hat, and I now move a vote of thanks for him by the Texas State Medical Association, of which he is a member. The law will save thousands of lives. It is of so vast importance that at first sight its far-reaching effects can not be appreciated. It will be the means of preventing diseases that carry off more lives than all the yellow fever, small pox and cholera combined. The public have scarcely a suspicion of the danger that lurks in upholstered cars, and sleeping apartments in public houses. Consumption alone kills 150 where yellow fever kills one, and the cars and hotels are nearly always infected with it, to say nothing of diphtheria, etc. Dr. Tabor has thus, by securing this law, signalized his administration by the most pronounced advance in sanitary reform within the century. He is certainly the right man in the right place, and should be kept there the balance of his life.

